# Subclinical leaflet thrombosis in Ozaki procedure

**DOI:** 10.1093/icvts/ivaf051

**Published:** 2025-03-01

**Authors:** Mathieu van Steenberghe, Francois Perret, Patrick O Myers, Gregory Khatchatourov

**Affiliations:** Cardiovascular Surgery Unit, Cecil Clinic, Lausanne, Switzerland; Cardiovascular Surgery Unit, Hôpitaux Universitaires de Genève, Geneva, Switzerland; Cardiology Unit, Cecil Clinic, Lausanne, Switzerland; Cardiovascular Surgery Unit, La Tour Hospital, Geneva, Switzerland; Cardiovascular Surgery Unit, Cecil Clinic, Lausanne, Switzerland

**Keywords:** subclinical leaflet thrombosis, Ozaki procedure, endothelialization, glutaraldehyde, anticoagulation

## Abstract

Aortic valve reconstruction with autologous glutaraldehyde-fixed pericardium (Ozaki procedure) represents an alternative to conventional prosthetic valve replacement, allowing excellent haemodynamic outcomes. We report two cases of subclinical leaflet thrombosis (SLT) at 12 and 23 months of follow-up. Anticoagulation was initiated, and later echocardiography showed haemodynamic and mobility improvement. SLT is well documented for bioprosthetic valve. To our knowledge, this is the first report for Ozaki procedure in an adult population. Glutaraldehyde is known for cytotoxicity, and partial endothelialization can be responsible for thrombosis, creating favourable conditions for later endocarditis and degeneration. Anticoagulation should be recommended postoperatively for the first 3 months with control at 6 months. Finally, alternative treatment to glutaraldehyde should be investigated.

## INTRODUCTION

The Ozaki procedure is a promising option for aortic valve replacement using autologous glutaraldehyde-fixed pericardium. The reported complications are endocarditis and aortic insufficiency (AI) [1].

Subclinical leaflet thrombosis (SLT) is documented for bioprosthesis prosthesis in surgical aortic valve replacement (SAVR) and transcatheter aortic valve implantation (TAVI) [2, 3].

We report two cases of SLT after Ozaki procedure and histological analysis of a neo valve of a third patient reoperated for AI.

## CASES PRESENTATION

**Figure 1:**
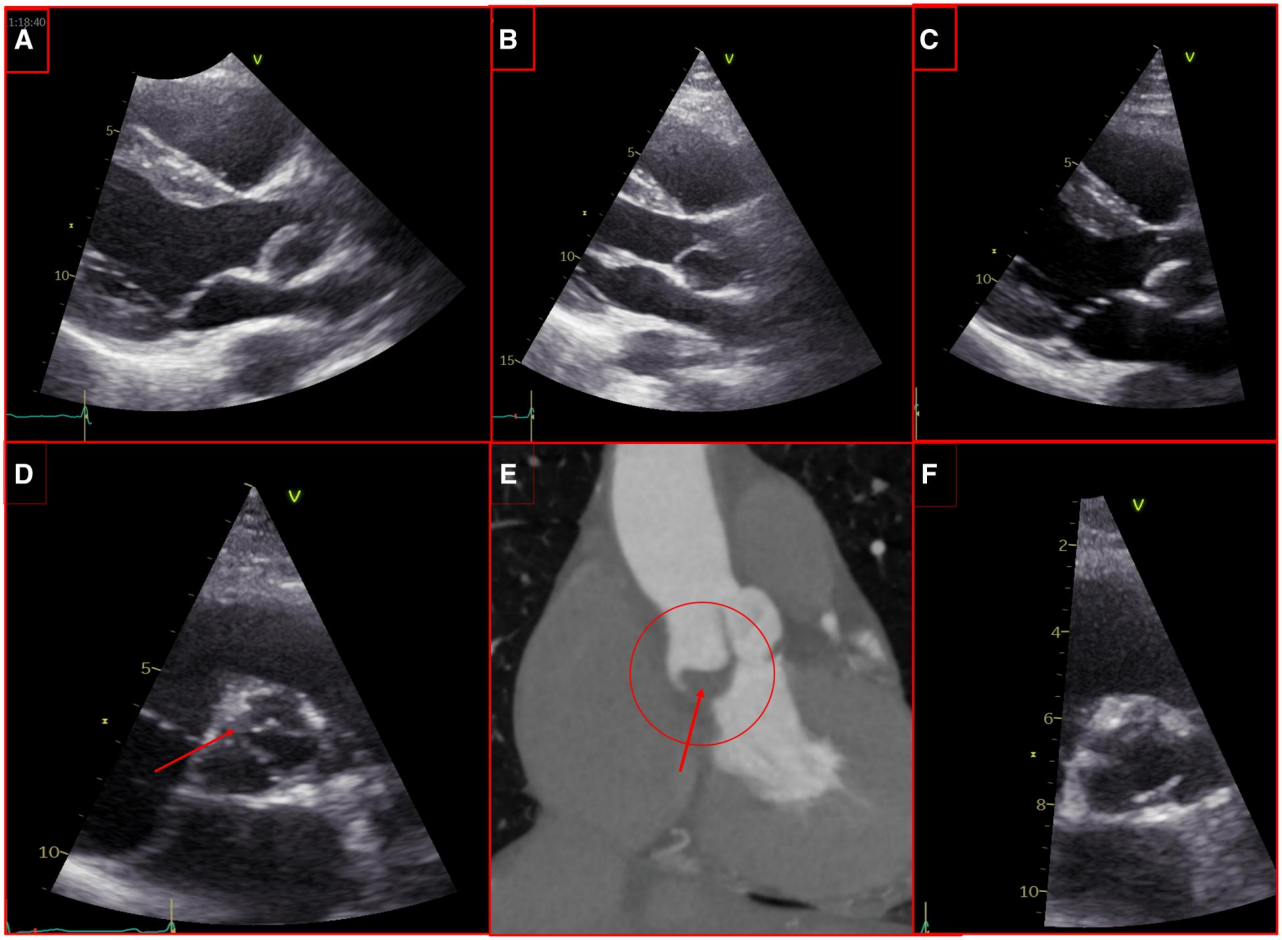
(**A**–**C**): First patient TTE: (**A**): at 12 months: leaflet thickening. (**B**): 4 months after anticoagulation: leaflet thickness restoration. (**C**): at 59 months: rethickening. Second patient TTE and Ct.: (**D**, **E**): TTE and CT at 24 months: thickening and thrombosis (red arrow). (**F**): TTE 4 months after anticoagulation: normal thickness.

The first patient is a 38-year-old gentleman who had an Ozaki procedure for severe AI in a bicuspid valve. As recommended by Ozaki *et al.*, the pericardium was treated with 10 min of glutaraldehyde with three successive baths of saline of 6 min. The patient received 3 months of antiaggregation with aspirin after surgery as recommended by Ozaki *et al.* Immediate postoperative transthoracic echocardiography (TTE) showed an adequate reconstruction, stable at 6 months follow-up. One year after surgery, the TTE showed attenuated mobility and moderate thickening of the non-coronary leaflet without significant stenosis and trivial regurgitation (Fig. 1A). After 4 months of anticoagulation with Vitamin K antagonist (VKA), the leaflet showed normal mobility and thickness (Fig. 1B). VKA was stopped after 12 months. Four months later, the leaflet showed re-thickening, and Apixaban (NOACs) was started. Two months later, the TTE showed reduction of thickening. After 28 months of NOACs, the leaflet was thicker without stenosis or regurgitation, and VKA was restarted. The latest follow-up, 3 months later, did not show improvement, suggesting leaflet fibrosis (Fig. 1C).

The second patient is a 66-year-old woman who had the same procedure for severe stenosis and the same antiaggregation policy after surgery. Immediate and 6 months postoperative TTE were normal. At 24 months, in asymptomatic patient, TTE showed important thickening and reduced mobility of the right coronary leaflet, with decrease of orifice area (OA) (Fig. 1D). CT scan confirmed these findings (Fig. 1E). VKA were introduced. Four months later, the neo-valve showed return to normal thickness, mobility and OA. At this time, anticoagulation with NOACs was introduced for patient non-compliance. After 8 months of NOACs, neo-valve function was excellent with no thickening.

**Figure 2:**
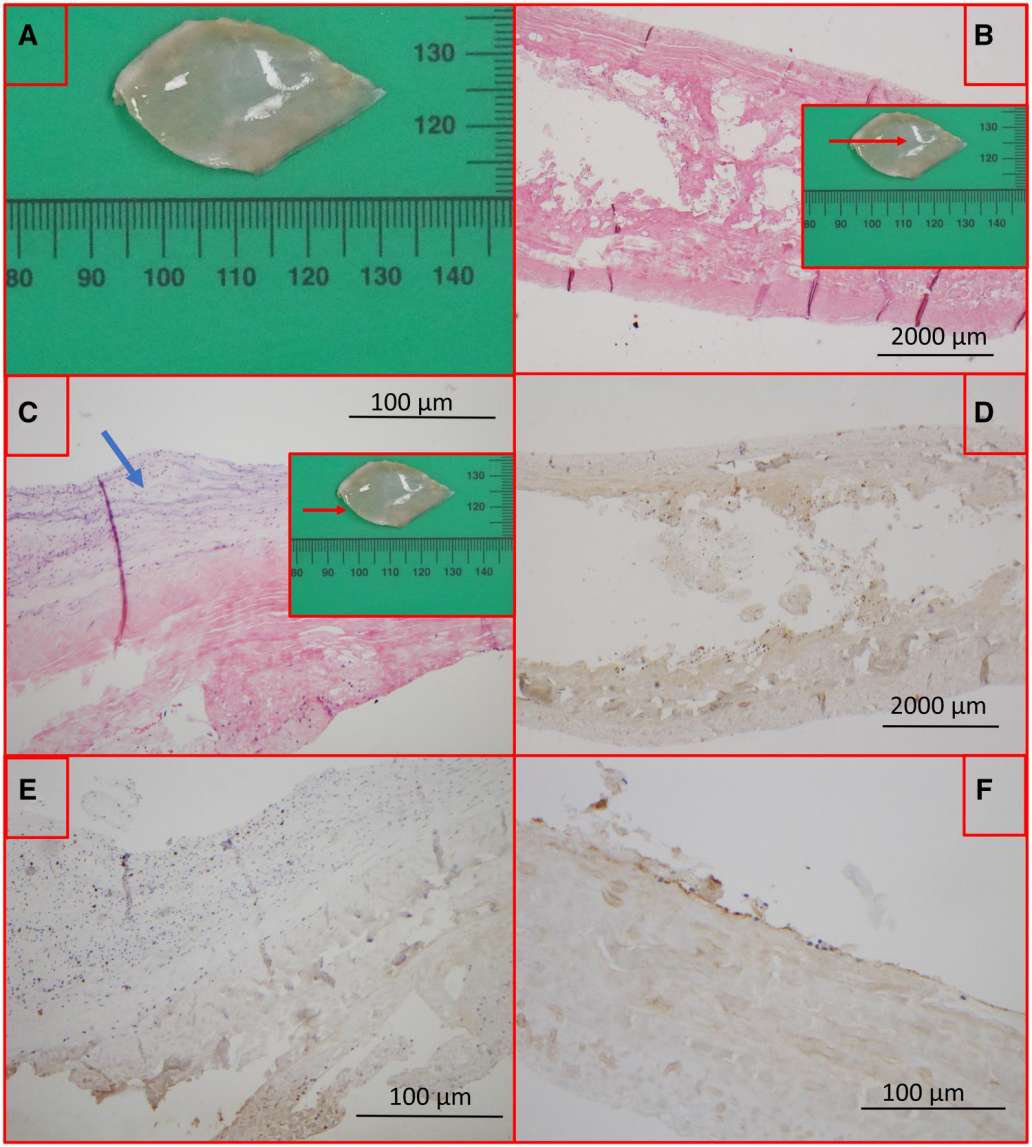
(**A**): Macroscopy of the removed neoleaflet. (**B**, **C**): leaflet haematoxylin and eosin staining (respectively at ×40 and ×100). Some fibrosis at the centre (**B**), elastofibrosis at the edge (**C**) (blue arrow). (**D**): low histiocyte (CD68) staining (×40). (**E**): low lymphocytes (CD3) staining (×100). (**F**): low endothelial (CD31) staining (×100).

To better understand this phenomenon specifically in the Ozaki procedure, we report histology analysis of neo leaflet resected for severe AI 3.5 months postoperatively due to suture line dehiscence. The patient received the same antiaggregation policy as the two previous cases. Histology (Fig. 2) showed low inflammatory reaction, no calcific remodelling and focal surface endothelialization with slight elastofibrosis.

## DISCUSSION

### Prevalence

SLT is defined as incidental finding characterized by a thin layer of thrombus covering the aortic aspect of the leaflet on echocardiography or computed tomography. It affects motion of the leaflets. The phenomenon occurs frequently in bioprosthetic aortic valves, more commonly in TAVI than in SAVR (at 30 days: 13% vs 5%) and could be responsible for early valve degeneration and can be associated with strokes [2, 3].

In our Ozaki cohort, SLT was incidentally diagnosed by TTE on three patients among 120 (a third patient was diagnosed, but imaging data were incomplete and not detailed in this report), showing a low prevalence. We did not observe any stroke, TIA or early valve degeneration.

### Mechanisms

Factors leading to SLT are (i) haemodynamic factors: altered flow dynamics (low coronary washout-sinus flow), altered valve geometry after implantation, (ii) surface factors: damage to the pericardial leaflet, native immune response with leaflet deterioration and lack of endothelialization during the first period after implantation and (iii) haemostatic factors [4, 5]. It results in adhesion of platelets and activated leukocytes that promotes inflammation, thrombosis and fibrosis [5].

Why can SLT occur after an Ozaki procedure, while the reconstruction respects native geometry and uses non-immunogenic tissue? We suggest first, related to haemodynamic factors: a large surface of pericardium is inserted, potentially creating altered flow in the sinuses (see Supplemtentary reference 6). Second, related to surface factors: while pericardium is not subject to immune rejection, partial endothelialization as shown could contribute to favourable condition for thrombosis and later degeneration. In this aspect, the cytotoxicity of glutaraldehyde could be incriminated (see Supplementary reference 7), and our research group then works on other crosslinkers to improve fast endothelialization.

### Prevention and treatment

Prevention and treatment after bioprosthetic valve replacement are recommended for further improvement in valve haemodynamics and structural maintenance and clinical outcome. ESC/EACTS guidelines recommend 3–6 months of dual antiplatelet therapy followed by single therapy, to prevent SLT after TAVI and either low-dose aspirin or a VKA for the first 3 months after SAVR [5] (Supplementary reference 8).

Ozaki *et al.* recommend only single antiplatelet therapy during 3 months. Some centres, particularly paediatric centres, modified this policy by adding anticoagulation or anticoagulation alone during the first 3 months (see Supplementary references 9 and 10).

Our experience with a prevention of 3 months of antiplatelet therapy alone following surgery shows SLT in Ozaki can occur in the adult population while anticoagulation restores leaflet mobility. But SLT can reoccur after adequate treatment as described for SAVR thrombosis, leading to irreversible pericardium alterations, suggesting aggressive prevention may be useful [5]. Based on this experience, we have adopted an initial prevention period of 3 months of anticoagulation after surgery with echocardiographic control at 3 and 6 months and, if SLT arises, to pursue anticoagulation after resolution to avoid risks of later degeneration.

## CONCLUSION

SLT occurs in all forms of pericardial aortic valve replacement and reconstruction, Ozaki, TAVI and SAVR, although it seems at a low incidence for Ozaki. Anticoagulation can restore leaflet mobility. We recommend initial prevention with anticoagulation, anticoagulation if SLT occurs and continuation of treatment after resolution to avoid risks of later degeneration.

## Supplementary Material

ivaf051_Supplementary_Data

## Data Availability

The data underlying this article will be shared on reasonable request to the corresponding author.

## CLINICAL REGISTRATION NUMBER

The study was conducted in compliance with the ethical requirements of Swiss law on the quality control of surgical procedures (RO 1995 1328, Loi fédérale du 18 mars 1994 sur l'assurance-maladie [LAMal] article 58). Patients were informed of the procedure and required to sign an informed consent for surgery. Individual written consent to publish their study data was also obtained.
