# Supplementary material for: Subclinical leaflet thrombosis in Ozaki procedure
Source: Interdiscip Cardiovasc Thorac Surg. 2025 Mar 1;40(3):ivaf051. doi: 10.1093/icvts/ivaf051 (PMC11913325; doi:10.1093/icvts/ivaf051)
Supplement: ivaf051_Supplementary_Data [file ivaf051_supplementary_data.docx]

**Supplementary Material**

**Supplementary References**

6/Aortic Valve Reconstruction With Autologous Pericardium Versus a Bioprosthesis: The Ozaki Procedure in Perspective. Unai S et al. J Am Heart Assoc. 2023 Jan 17;12(2).

7/Glutaraldehyde treatment elicits toxic response compared to decellularization in bovine pericardium. Umashankar PR et al. Toxicol Int. 2012;19:51.

8/Subclinical leaflet thrombosis and antithrombotic therapy post-TAVI: An LRT substudy. Bhogal S, et al. Int J Cardiol. 2023 Jan 15;371:305-311.

9/ Aortic valve neocuspidalization in paediatric patients with isolated aortic valve disease: early experience. Polito et al.  *Interactive CardioVascular and Thoracic Surgery*, Volume 32, Issue 1, January 2021, Pages 111–117.

10/ Congenital aortic and truncal valve reconstruction using the Ozaki technique: Short-term clinical results Baird, Christopher W. et al. The Journal of Thoracic and Cardiovascular Surgery, Volume 161, Issue 5, 1567 - 1577
